# Dysregulated Hepatic Methionine Metabolism Drives Homocysteine Elevation in Diet-Induced Nonalcoholic Fatty Liver Disease

**DOI:** 10.1371/journal.pone.0136822

**Published:** 2015-08-31

**Authors:** Tommy Pacana, Sophie Cazanave, Aurora Verdianelli, Vaishali Patel, Hae-Ki Min, Faridoddin Mirshahi, Eoin Quinlivan, Arun J. Sanyal

**Affiliations:** 1 Div. of Gastroenterology, Hepatology and Nutrition, Dept. of Internal Medicine, Virginia Commonwealth University School of Medicine, Richmond, VA, 23298, United States of America; 2 Biomedical Mass Spectrometry Laboratory, General Clinical Research Center, University of Florida, Gainesville, FL, United States of America; CIMA. University of Navarra, SPAIN

## Abstract

Methionine metabolism plays a central role in methylation reactions, production of glutathione and methylarginines, and modulating homocysteine levels. The mechanisms by which these are affected in NAFLD are not fully understood. The aim is to perform a metabolomic, molecular and epigenetic analyses of hepatic methionine metabolism in diet-induced NAFLD. Female 129S1/SvlmJ;C57Bl/6J mice were fed a chow (n = 6) or high-fat high-cholesterol (HFHC) diet (n = 8) for 52 weeks. Metabolomic study, enzymatic expression and DNA methylation analyses were performed. HFHC diet led to weight gain, marked steatosis and extensive fibrosis. In the methionine cycle, hepatic methionine was depleted (30%, p< 0.01) while s-adenosylmethionine (SAM)/methionine ratio (p< 0.05), s-adenosylhomocysteine (SAH) (35%, p< 0.01) and homocysteine (25%, p< 0.01) were increased significantly. SAH hydrolase protein levels decreased significantly (p <0.01). Serine, a substrate for both homocysteine remethylation and transsulfuration, was depleted (45%, p< 0.01). In the transsulfuration pathway, cystathionine and cysteine trended upward while glutathione decreased significantly (p< 0.05). In the transmethylation pathway, levels of glycine N-methyltransferase (GNMT), the most abundant methyltransferase in the liver, decreased. The phosphatidylcholine (PC)/ phosphatidylethanolamine (PE) ratio increased significantly (p< 0.01), indicative of increased phosphatidylethanolamine methyltransferase (PEMT) activity. The protein levels of protein arginine methytransferase 1 (PRMT1) increased significantly, but its products, monomethylarginine (MMA) and asymmetric dimethylarginine (ADMA), decreased significantly. Circulating ADMA increased and approached significance (p< 0.06). Protein expression of methionine adenosyltransferase 1A, cystathionine β-synthase, γ-glutamylcysteine synthetase, betaine-homocysteine methyltransferase, and methionine synthase remained unchanged. Although gene expression of the DNA methyltransferase *Dnmt3a* decreased, the global DNA methylation was unaltered. Among individual genes, only HMG-CoA reductase (*Hmgcr*) was hypermethylated, and no methylation changes were observed in *fatty acid synthase* (*Fasn)*, *nuclear factor of kappa light polypeptide gene enhancer in B-cells 1* (*Nfκb1)*, *c-Jun*, *B-cell lymphoma 2 (Bcl-2) and Caspase 3*. NAFLD was associated with hepatic methionine deficiency and homocysteine elevation, resulting mainly from impaired homocysteine remethylation, and aberrancy in methyltransferase reactions. Despite increased PRMT1 expression, hepatic ADMA was depleted while circulating ADMA was increased, suggesting increased export to circulation.

## Introduction

Nonalcoholic fatty liver disease (NAFLD) is the most common cause of chronic liver disease in many parts of the world [1]. It is closely linked to the associated presence of insulin resistance and the metabolic syndrome [2, 3]. The clinical-histological phenotype of NAFLD extends from nonalcoholic fatty liver (NAFL) to nonalcoholic steatohepatitis (NASH). NAFLD is associated with increased mortality [4]; cardiovascular-, liver- and cancer-related deaths account for this excess mortality [5, 6]. The pathogenesis of NAFLD is only incompletely understood.

Methionine is an essential amino acid and plays a key role as a regulator of several cellular functions [7, 8]. Its metabolism takes place mainly in the liver [9]. It is converted to s-adenosylmethionine (SAM), the primary source of methyl groups, required for transmethylation reactions and needed for the synthesis of phosphatidylcholine, creatine, sarcosine and methylarginines [10, 11]. Enzymes that catalyze DNA methylation also utilize the methyl group from SAM. Demethylation of SAM converts it sequentially to s-adenosylhomocysteine (SAH) and homocysteine [12]. Homocysteine can be remethylated to methionine or used for transsulfuration reactions that generate glutathione [13]. Hyperhomocysteinemia is associated with the metabolic syndrome and NAFLD [14–16] and has been associated with oxidative stress, inflammation, unfolded protein response, cell death and increased cardiovascular risk [17–20]. These pathways are all currently believed to be important in the pathogenesis of NASH [21]. The mechanism(s) by which hyperhomocysteinemia develops in NAFLD is not well understood.

There are only limited data on the impact of diet-induced obesity and development of NAFLD on methionine metabolism and how that impacts levels of homocysteine, ability to replenish glutathione, form biologically active methylarginines and the methylation status of key targets involved in the pathogenesis of NASH. The existing literature has focused mainly on early time points (3–12 weeks) following a high-fat diet and most have focused on selected metabolic pathways [22–25]. While some have shown SAM depletion [22, 23], others have been unable to demonstrate such changes at these early time points when steatosis is present but where the disease has not progressed to bridging fibrosis or cirrhosis [24, 25]. Thus, the status of hepatic methionine metabolism in diet-induced obesity and advanced NAFLD remains to be clarified.

The objective of the current study was to define the status of methionine metabolic pathways and production of key downstream metabolites in diet-induced obesity and NAFLD using a combined metabolomic and molecular approach. The study was performed in a mouse model of NAFLD which progressed to advanced fibrosis after 52 weeks of high-fat high-cholesterol (HFHC) diet.

## Methods

### Mice and diets

Female 129S1/SvlmJ;C57Bl/6J mice were kindly provided by Dr. Sandra Erickson (UCSF, CA) and kept on a 12:12-h light-dark cycle. At 10 weeks of age, the mice were randomly assigned to two groups: chow (n = 6) and HFHC diet group (n = 8). The chow group was fed a standard chow diet (Teklad 7012, Harlan) with 17% of energy derived from fat, 58% from carbohydrates, and 25% from protein; HFHC group received a western diet (Teklad 88137, Harlan) with 42% of energy derived from fat, 43% from carbohydrate, and 15% from protein. Food and water were provided ad libitum for 52 weeks until sacrifice. At the end of the feeding period, the animals were fasted for 12 hours, euthanized by CO2, and body weights determined. Blood was collected through heart puncture, allowed to clot, and serum obtained by centrifugation at 3,000 rpm for 15 minutes at 4°C. Livers were harvested, weighed, and dissected; a portion of fresh tissue was fixed in 10% buffered formalin and remaining tissues were snap-frozen in liquid nitrogen. Serum and liver samples were stored at -80°C until RNA and protein isolation or biochemical analyses. All animal experiments were approved by Institutional Animal Care and Use Committee of Virginia Commonwealth University.

### Serum biochemistry profile

Serum measurements of aspartate aminotransferase (AST), alanine aminotransferase (ALT), alkaline phosphatase (ALP), gamma-glutamyltransferase, bilirubin, cholesterol, high-density lipoprotein (HDL), low-density lipoprotein (LDL), and triglycerides were performed in the clinical chemistry laboratories of the author’s institution using established commercially available methods.

### Histology

Liver sections were fixed in 10% formalin. Tissues were embedded in paraffin blocks and stained with hematoxylin and eosin (HE) and Masson’s trichrome stain using standard commercially available methods. Steatosis, inflammation and fibrosis were quantified separately.

### RNA extraction and real-time reverse transcription PCR

Primers were designed using Beacon Designer software (Bio-Rad Laboratories, Inc.), verified by BLAST search and prepared by the Nucleic Acid Core facility at Virginia Commonwealth University (S1 Table). Total RNA was isolated from the liver with a commercial RNA isolation kit (Trizol, Invitrogen) in accordance with the manufacturer’s instructions (Invitrogen, CA) and purified with RNase-free DNase. cDNA was synthesized from 4 μg of total RNA by using Maloney-murine leukemia virus reverse transcriptase and oligo(dT) primers and subjected to PCR by the manufacturer's instructions. Quantitative RT-PCR was performed by SYBR Green PCR master mix (BioRad, Hercules, CA) on an ABI Prism 7300 Sequence Detection System as previously described [26]. GAPDH was used as the endogenous normalizer. Cycle threshold (Ct) values were normalized to GAPDH and comparative quantification of target mRNA was done by ΔΔCt method using integrated software with Stratagene Mx3000P QPCR system.

### Protein extraction and western blot analysis

Mouse liver tissues were lysed using RIPA lysis buffer (Sigma, St. Louis, MO) and subjected to sonication on ice with a Sonicator cell disrupter, Model 100 Sonic Dismembrator (Thermo Fisher Scientific Inc., Waltham, MA). Cell lysates were centrifuged at 12,000 g x 15mins, supernatants collected, and separated using 4%–12% NuPAGE Novex Bis-Tris Mini Gels (Invitrogen) and transferred to a nitrocellulose membrane for 1 hr at 40V using a Western blot apparatus (Invitrogen). After overnight incubation with primary antibody, membranes were washed, incubated with HRP-conjugated secondary antibodies (Pierce Biotechnology, Inc., Rockford, IL) and detected using the SuperSignal chemiluminescence kit (Pierce). The signal capture and protein level analyses were performed as described before [27].

### Standards and stable isotope labeled standards

Asymmetric-dimethylarginine (ADMA) and symmetric-dimethylarginine (SDMA) were purchased from Axxora LLC (San Diego, CA). Methylarginine (NG-mono-methyl-arginine) was purchased from Acros Organics (New Jersey). S-adenosyl-homocysteine (SAH) was purchased from Cayman Chemical Co. (Ann Arbor, MI). S-adenosyl-methionine (SAM) was purchased from New England Biochemicals (Ipswich, MA). The remaining standards were purchased from Sigma-Aldrich (St Louis, MO).

The following isotopically labeled standards [N,N-methyl-^2^H_6_]dimethylglycine, [2,3,3-^2^H_3_]serine, [U-^13^C]methionine, [1,2-^13^C_2_]glycine, [guanido-^15^N_2_]arginine, [^2^H_11_]betaine, [methyl-^2^H_3_]sarcosine, [methyl-^2^H_3_]creatine, [methyl-^2^H_3_]creatinine, 2,2-^2^H_2_]guanidineacetic acid, [2,3,3,4,4,5,5-^2^H_7_]ornithine, [trimethyl-^2^H_9_]choline chloride and [3,3,4,4-^2^H_4_]cystathionine were purchased from Cambridge Isotopes (Andover, MA). [methyl-^2^H_3_]SAM was purchased from CDN Isotopes (Pointe-Claire, Quebec, Canada). [3,3’,4,4’-^2^H_4_]SAH was purchased from Cayman Chemical Co. (Ann Arbor, MI). [U-13C]methionine sulfoxide was synthesized by oxidizing [U-13C]methionine with hydrogen peroxide. [^2^H_7_]methylarginine, [^2^H_7_]ADMA and [^2^H_7_]SDMA were each synthesized from [2,3,3,4,4,5,5-^2^H_7_]ornithine. A stock internal standard solution was prepared by diluting the isotopically labeled compounds, except [^2^H_3_]SAM and [^2^H_4_]SAH, in water. The [^2^H_3_]SAM and [^2^H_4_]SAH stock solutions were prepared freshly, by dissolving the solid compounds in 100 mM HCl, and mixing at the appropriate concentration with the other internal standards.

### Measurement of one-carbon metabolite concentrations

Just prior to homogenization, acetic acid (100 mmol/L) containing the labeled internal standards was added to the liver sample (20 mg each). The volume of internal standard mixture added was equal to 10 μl per mg of weighed tissue (200 μl per 20 mg tissue). The sample was then homogenized using a Pellet pestel (Kontes).

To precipitate proteins, 150 μl ice cold methanol was added to 50 μl homogenate. After vortexing to mix, the sample was centrifuged at 20,000 g for 5 min. The supernatant was then analyzed by LC-MS/MS using a Thermo-Finnigan Quantum Ultra in SRM mode. The ratio of each analyte to its internal standard was calculated and the analyte concentration was determined by comparing against standard calibration curves.

### Measurement of homocysteine, cysteine, glutathione and cysteinyl-glycine concentrations

Total homocysteine, cysteine, glutathione and cysteinyl-glycine concentrations were measured after reducing to free thiols and derivatizing by HPLC with fluorescence detection as previously described [28].

### Measurement of phosphatidylethanolamine and phosphatidylcholine concentrations

The liver tissue samples were weighed, pulverized with the CP02 CryoPrep Dry Pulverization System (Covaris), and resuspended in ice-cold methanol containing 0.1% butyl-hydroxy-toluene (BHT) in a concentration of 100 mg/ml. Phospholipids were extracted using a modified Folch lipid extraction [29] performed on a Hamilton Microlab Star robot. Samples were spiked with known amounts of non-endogeneous synthetic internal standards as described by Ståhlman et al. [30]. After lipid extraction, samples were reconstituted in chloroform:methanol (1:2, v/v). The extracts were stored at -20°C prior to MS analysis.

The lipid extracts were analyzed on a hybrid triple quadrupole/linear ion trap mass spectrometer (QTRAP 5500) equipped with a robotic nanoflow ion source (NanoMate HD) according to Ståhlman and colleagues [30]. Molecular lipids were analyzed in both positive and negative ion modes using multiple precursor ion scanning (MPIS) and neutral loss (NL) based methods [31, 32]. Lipids were normalized to their respective internal standard and the tissue weight.

### Determination of percentage deoxycytidine methylation

Genomic DNA was extracted from 10 mg of liver using a DNeasy Blood and Tissue Kit (Qiagen, Germantown, MD) according to the manufacturer’s instructions. After adding biosynthetic [^15^N_3_]dC and [^15^N_3_]5mdC internal standards [33] to the liver DNA, the DNA was digested to nucleosides [33, 34]. The concentrations of deoxycytidine and 5-methyldeoxycytidine (5mdC) were determined by LC-MS/MS [33]. Percentage deoxycytidine methylation (% 5mdC) was calculated as: % 5mdC = [5mdC] / ([dC] + [5mdC]) x 100. Inter- and intra-assay variation (relative standard deviation; n = 6) for the assay was < 2.5%.

### Determination of relative 5-hydroxymethyl-2’-deoxycytidine concentrations

Labeled [^15^N_3_] standards for 5-hydroxymethyl-2’-deoxycytidine (5hmdC) analysis were biosynthesized from [^15^N_3_]DNA and formaldehyde, after which it was digested to nucleosides. The resulting [^15^N_3_]dC and [^15^N_3_]5hmdC was added to the samples of liver DNA, and the DNA digested to nucleosides and analyzed by LC-MS/MS [34]. The mass transitions used for 5hmdC and [^15^N_3_]5hmdC were 258→142 and 261→145, respectively. As we did not have an authentic 5hmdC standard for determining absolute 5hmdC concentrations, relative 5hmdC concentration (relative to dC) were calculated from the analyte and internal peak areas: Relative 5hmdC conc. = (5hmdC / [^15^N_3_]5hmdC) ÷ (dC / [^15^N_3_]dC). These are arbitrary units. The intra-assay variation (relative standard deviation; n = 6) for the assay was < 6%.

### DNA methylation status of HMGCR, FASN, NFkB1, c-Jun, Bcl-2 and Caspase 3

DNA methylation analyses of promoter CpG islands for 3-hydroxy-3-methylglutaryl-coenzyme A reductase (*Hmgcr*) and fatty acid synthase (*Fasn*), nuclear factor of kappa light polypeptide gene enhancer in B-cells 1 (*Nfkb1*), *c-Jun*, B-cell lymphoma 2 (*Bcl-2*) and Caspase 3 targets were performed by EpiTect® Methyl II PCR arrays (Qiagen, Frederick, MD) using a methylation-sensitive and/or methylation-dependent restriction enzyme digestion, followed by SYBR Green real-time PCR detection. Using the ΔCt method, the relative amount of methylated and unmethylated DNA fractions were calculated by comparing the amount in each digest with that of a mock (no enzymes added) digest.

### Statistical analysis

The levels of specific metabolites in chow-fed versus HFHC diet-fed mice were compared using unpaired Student’s T-test or Kruskal Wallis analysis of variance as appropriate. Data are presented as the mean ± standard error of the mean (SEM). Given a standard deviation of 20% of the mean, a sample of 7 in each group was estimated to be able to detect a 25–30% change across groups with a power of 80%. A p value of < 0.05 was considered to be significant.

## Results

### Morphology, biochemical profile and histology

A total of 6 and 10 mice were given either chow diet or HFHC diet, respectively, for 52 weeks. Two mice died between week 32–40 and a total of 8 and 6 mice receiving HFHC or chow diet, respectively, survived to the end of study. Mice fed with HFHC diet significantly gained more body weight (50.5 g vs 36.7 g; p< 0.01) and liver weight (2.4 g vs 1.3 g; p< 0.05) than mice fed with chow diet at 52 weeks (Table 1). HFHC diet also led to significantly increased serum aminotransferases, cholesterol, triglycerides, HDL, LDL and HOMA-IR. Histological examination of the liver confirmed the presence of steatosis, involving over 75% of the liver in mice fed a HFHC diet (S1 Fig). The steatosis was panacinar and included both macrovesicular and small droplet steatosis. Sheets of cells with small droplet steatosis, predominantly centrilobular in distribution, were seen. There were also scattered foci of inflammatory cells and extensive pericellular fibrosis (S1 Fig).

**Table 1:** Morphological and biochemical profile at 52 weeks of chow and HFHC diet.

	Chow (n = 6)	HFHC (n = 8)	P-value
Body weight (gram)	36.7 ± 2.4	50.5 ± 3.0	<0.05
Liver weight (gram)	1.3 ± 0.1	2.4 ± 0.4	<0.05
Insulin (mU/l)	48.4 ± 5.1	65.5 ± 8.7	<0.05
HOMA-IR	43.6 ± 5.8	61.78 ± 8.2	<0.05
AST (unit/L)	220.8 ± 57.6	628.4 ± 83.6	<0.01
ALT (unit/L)	58 ± 6.0	560.5 ± 127.5	<0.01
ALP (unit/L)	40.5 ± 8.9	132.0 ± 26.0	<0.05
GGT (unit/L)	<0.5	<0.5	NA
Bilirubin (mg/dl)	<0.1	<0.1	NA
Cholesterol (mg/dl)	128.8 ± 10.6	337.4 ± 40.2	<0.01
HDL (mg/dl)	54.3 ± 4.4	105.8 ± 6.5	<0.01
LDL (mg/dl)	56.0 ± 6.3	209.8 ± 33.3	<0.01
Triglyceride (mg/dl)	92.7 ± 1.1	110.9 ± 2.6	<0.05

Data are represented as mean ± SEM. aspartate aminotransferase, AST; alanine aminotransferase, ALT; alkaline phosphatase, ALP; gamma-glutamyltransferase, GGT; high-density lipoprotein, HDL; high-fat high-calorie, HFHC; homeostasis model assessment of insulin resistance, HOMA-IR; low-density lipoprotein, LDL

### HFHC diet induces multiple changes in the methionine cycle

#### A: Methionine depletion, increased SAM/methionine ratio, SAH elevation and excess homocysteine in the liver with HFHC diet

**Fig 1:**
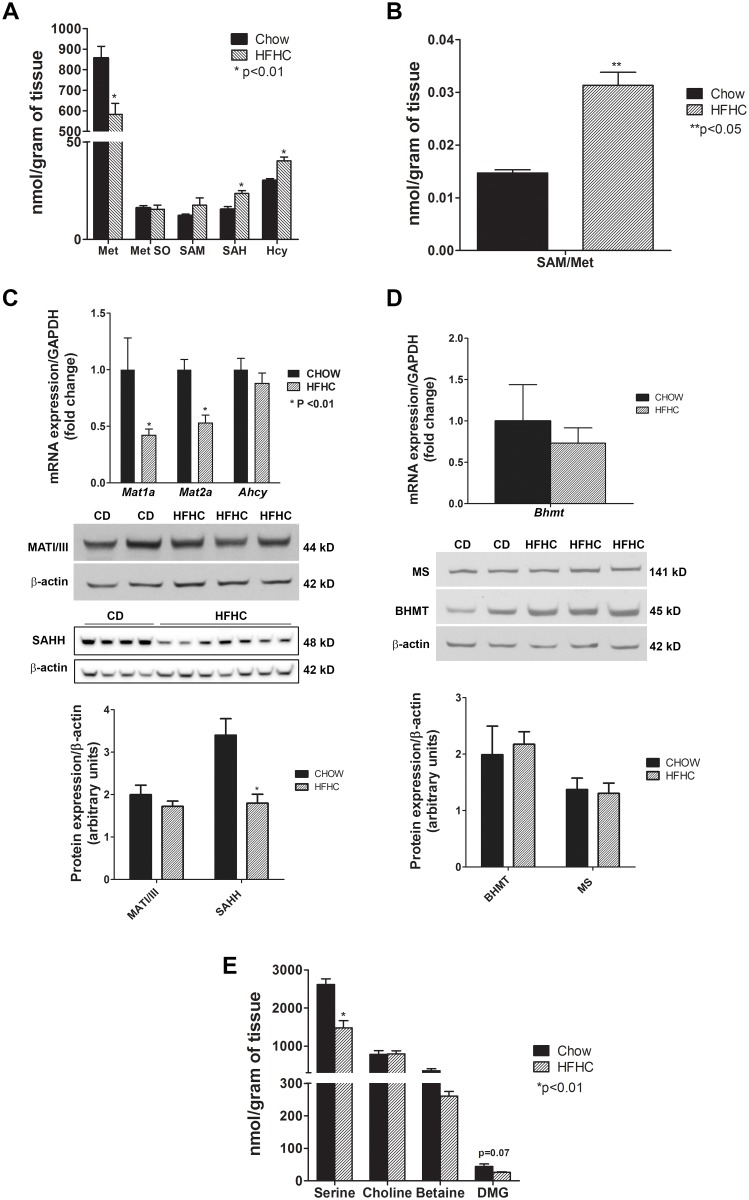
Methionine cycle: hepatic methionine depletion and homocysteine accumulation in diet-induced NAFLD. (A) HFHC diet for 52 weeks resulted in methionine (met) depletion (p< 0.01) and increased downstream products s-adenosylmethionine (SAM), s-adenosylhomocysteine (SAH) (p< 0.01) and homocysteine (Hcy) (p< 0.01) and (B) increased SAM/methionine ratio (p< 0.05), which is indicative of increased methionine utilization. Methionine sulfoxide (Met So) concentration remained unchanged. (C) The gene expression of methionine adenosyltransferase *Mat1a* and *Mat2a* was decreased (p< 0.01). However, the protein levels of MAT I/III, expressed solely by hepatic *Mat1a*, were relatively unchanged. Although *SAH hydrolase* (*Ahcy*) mRNA levels did not change, SAHH protein expression was decreased significantly (p< 0.01) which correlated with the increase in SAH levels but not the excess in Hcy. (D) The gene expression of *betaine-homocysteine methyltransferase* (*Bhmt*) and protein levels of BHMT and methionine synthase (MS), enzymes responsible for conversion of homocysteine to methionine, did not change. (E) Serine, the main one-carbon donor in the folate cycle needed for homocysteine remethylation, was depleted (p< 0.01). Whereas betaine levels trended down modestly, the decrease in dimethylglycine (DMG) almost approached significance (p = 0.07) that further indicates impairment of methionine reformation. Together, these findings suggest that homocysteine accumulation mainly results from impaired homocysteine remethylation to methionine. Data are represented as mean ± SEM.

The first step in the methionine cycle is demethylation of methionine to SAM via methionine adenosyltransferase (MAT). MAT is the product of two different genes, *Mat1a* and *Mat2a*. The former is expressed only in adult hepatocytes while the latter is expressed both in hepatic and extrahepatic tissues [35]. HFHC diet led to 30% depletion of methionine (p< 0.01) and a modest non-significant increase in SAM. The SAM/methionine ratio increased significantly (p< 0.05) (Fig 1A and 1B). To determine whether the observed decrease in methionine was due to its conversion to oxidative metabolites, methionine sulfoxide was also quantified. The levels of methionine sulfoxide did not change significantly (Fig 1A). Both the gene expressions of *Mat1a*, which encodes for MATI/III, and *Mat2a* were significantly decreased (Fig 1C). However, MAT I/III protein levels were relatively unchanged. The concentrations of SAH, the downstream product of SAM-derived transmethylation reactions, was elevated in HFHC group by 1.5 fold (p< 0.01) (Fig 1A). The protein levels of SAH hydrolase (SAHH), the enzyme that catalyzes the breakdown of SAH to homocysteine, were decreased significantly (p = 0.0022) (Fig 1C). On the other hand, the concentration of homocysteine was increased significantly (Fig 1A).

#### B: Metabolomic evidence of impaired remethylation of homocysteine to methionine

Homocysteine is remethylated to methionine via methionine synthase or via betaine-homocysteine methyltransferase. Methionine synthase activity requires serine-dependent activity of the folate cycle as a donor of methyl groups from methyltetrahydrofolate [36]. Despite a high homocysteine, methionine levels were low as noted above. The protein expression of methionine synthase remained unchanged on a HFHC diet (Fig 1D). However, there was a profound depletion of serine (p< 0.01) (Fig 1E), which is expected to impair the activity of the folate cycle. The levels of choline, the precursor of betaine, were not significantly altered. Whereas betaine levels trended down modestly, although non-significant, the levels of dimethylglycine, the downstream product of betaine-homocysteine methyltransferase (BHMT) activity, were even further decreased (p = 0.07) resulting in a decrease in dimethylglycine:betaine ratios. The gene and protein expression of BHMT was relatively unchanged with HFHC diet (Fig 1D).

### The transsulfuration pathway is preserved after a HFHC diet

**Fig 2:**
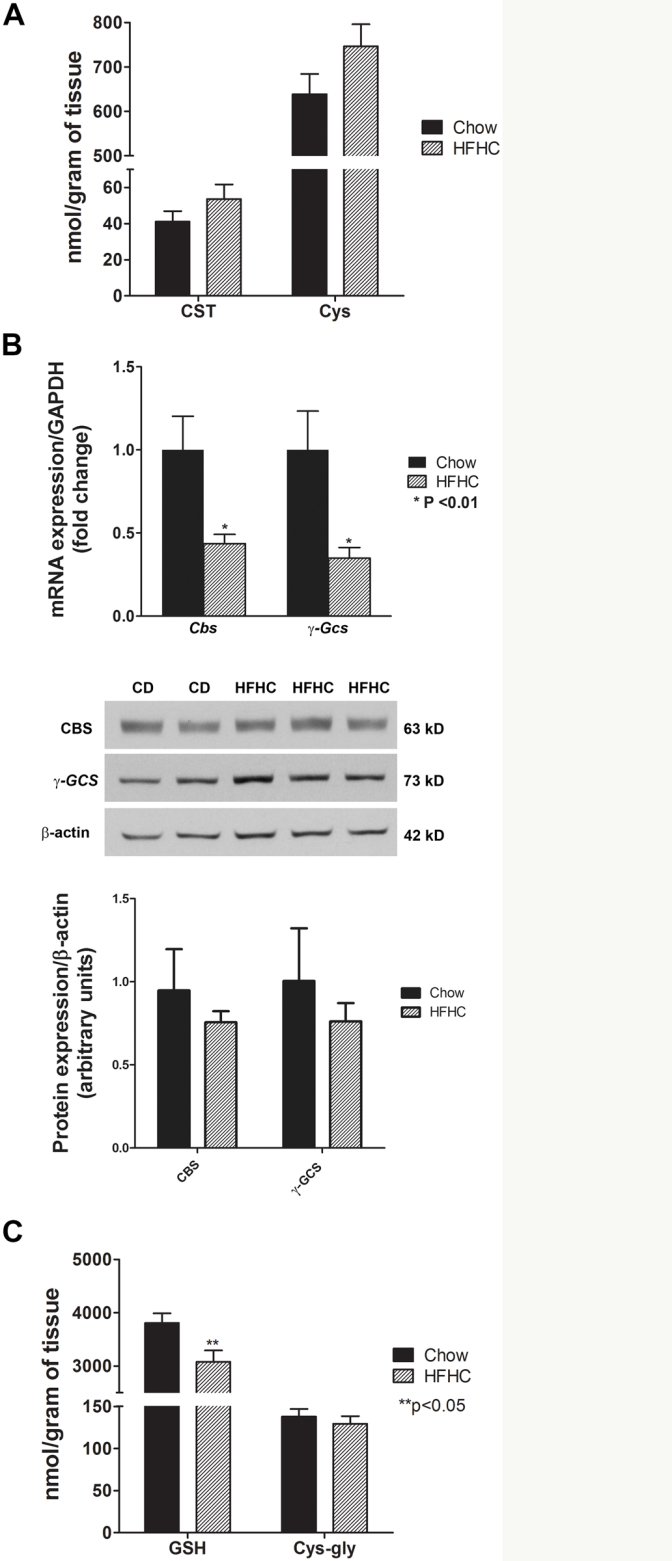
Transsulfuration pathway: depletion of serine limits the ability to replete glutathione in diet-induced NAFLD. (A) HFHC diet for 52 weeks resulted only in modest non-significant increase in cystathionine (CST) and cysteine (Cys) levels in spite of homocysteine accumulation and decrease in glutathione levels. (B) mRNA expression of *cystathionine β-synthase* (*Cbs*) and *γ-glutamylcysteine synthetase* (*γ-Gcs*) decreased but the protein levels of CBS and GCS did not significantly change. (C) Glutathione (GSH) was depleted (p< 0.05), likely as a result of oxidative stress, while cysteinyl-glycine, a catabolic product of GSH, remained unchanged (C). Data are represented as mean ± SEM.

Another mechanism to remove homocysteine is through the transsulfuration pathway by conversion of homocysteine to cystathionine by cystathionine β-synthase. Cystathionine is then converted to cysteine for GSH synthesis via γ-glutamylcysteine synthetase. There was a modest non-significant increase in the levels of cystathionine and cysteine in mice on the HFHC diet (Fig 2A). However, this was not proportional to the increase in homocysteine and the homocysteine:cystathionine and homocysteine:cysteine ratios trended downwards in obese mice with NAFLD. This could be influenced by the depletion of the substrate serine, which is also needed to convert cystathionine from homocysteine, as noted above. Both *cystathionine β-synthase* and *γ-glutamylcysteine synthetase* mRNA levels decreased significantly in mice with NAFLD (Fig 2B). The protein levels of these enzymes also trended down but these changes were not significant. There was a significant decrease in the glutathione levels (p< 0.05) (Fig 2C) which is an indication of oxidative stress. The levels of cysteinyl-glycine remained unchanged. The ratio of reduced to oxidized glutathione trended upward, suggesting enhanced formation of glutathione to replete glutathione stores.

### Differential changes in transmethylation pathways

**Fig 3:**
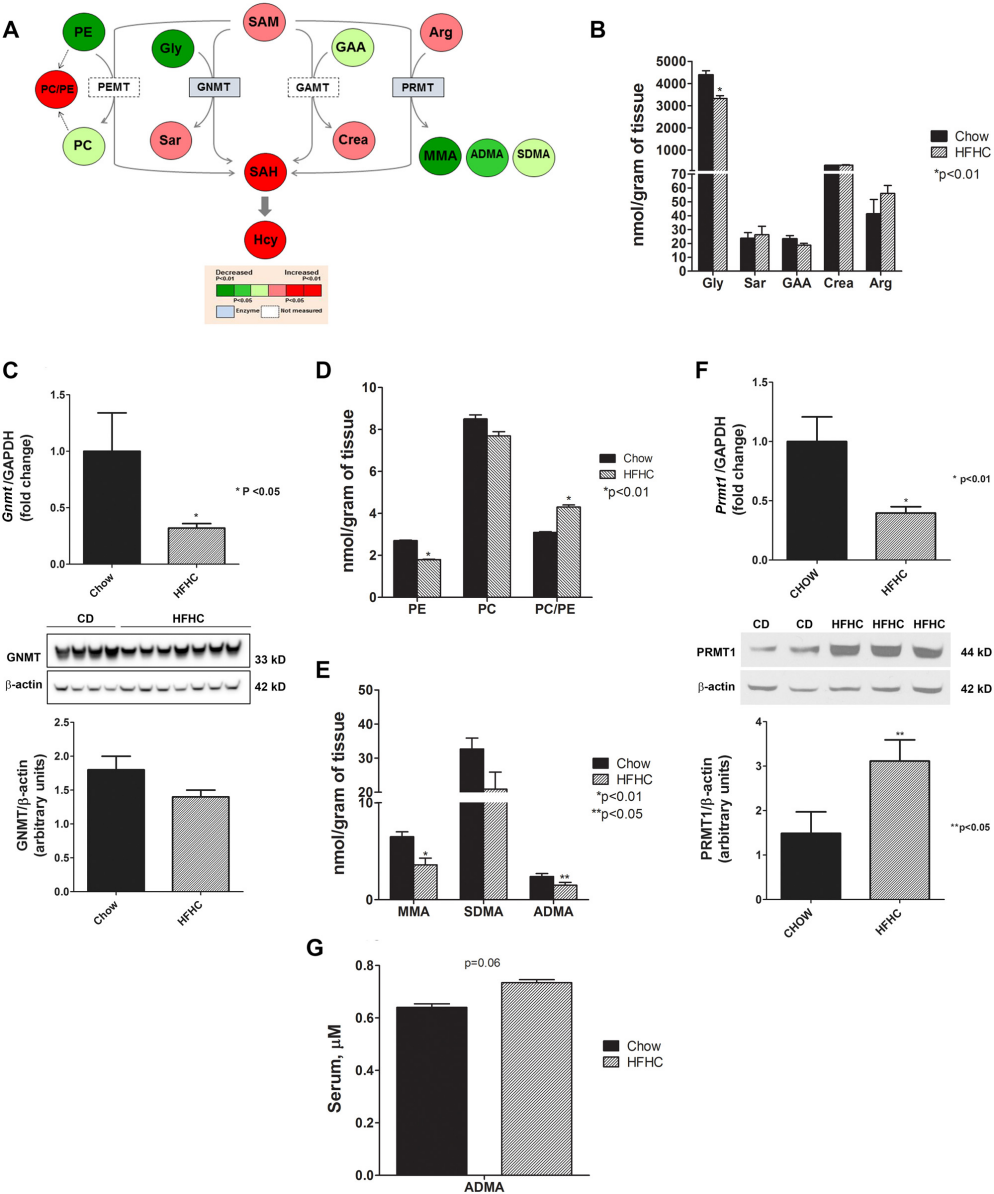
Transmethylation pathway: aberrancy in methyltransferase reactions in diet-induced NAFLD. (A) Heat map representation of substrates and methylated products of major SAM-derived transmethylation reactions (PEMT, GNMT, GAMT and PRMT) with HFHC diet for 52 weeks. (B) Levels of glycine, a substrate for GNMT, were decreased (p< 0.01) while the product sarcosine did not change between chow and HFHC group. (C) The gene expression of *Gnmt*, the most abundant methyltranserase in the liver, decreased significantly but only approached significance at the protein level (p = 0.06). (D) Levels of PE, the substrate for PEMT, was significantly reduced (p< 0.01) while PC trended down, resulting in a significant PC/PE ratio (p< 0.01), which suggests increase in PEMT activity There were no significant changes in the concentrations of guanidinoacetate and creatine, the substrate and product for GAMT, respectively. (E) The methylated products of PRMT activity, MMA and ADMA, were decreased significantly (p< 0.01 and p< 0.05) while the decrease in SDMA approached significance (p = 0.07). (F) The gene expression of *Prmt1*, mainly responsible for the methylation of MMA and ADMA, decreased (p< 0.01) but the protein levels of PRMT1 increased significantly (p< 0.05). (G) Circulating ADMA was increased toward significance (p = 0.06), suggesting increased export to the circulation. Data are represented as mean ± SEM. Legend: arginine (Arg); asymmetric dimethylarginine (ADMA); creatine (Crea); glycine (Gly); glycine N-methyltransferase (GNMT); guanidinoacetate (GAA); guanidinoacetate methyltransferase (GAMT); homocysteine (Hcy); monomethylarginine (MMA); phosphatidylcholine (PC); phosphatidylethanolamine (PE); phosphatidylethanolamine methyltransferase (PEMT); protein arginine methyltransferase (PRMT); s-adenosylhomocysteine (SAH); s-adenosylmethionine (SAM); sarcosine (Sar); symmetric dimethylarginine (SDMA).

Substrates and methylated products of major methyltransferase reactions were quantified: glycine and sarcosine via glycine N-methyltransferase (GNMT); phosphatidylethanolamine (PE) and phosphatidylcholine (PC) via phosphatidylethanolamine methyltransferase (PEMT); guanidinoacetate (GAA) and creatine via guanidinoacetate methyltransferase (GAMT); and arginine and monomethylarginine (MMA), symmetric dimethylarginine (SDMA) and ADMA via protein arginine methyltransferase (PRMT) (Fig 3A).

For the GNMT reaction, the concentration of glycine (the substrate) was significantly reduced while the concentration of sarcosine (the product) did not change significantly in mice with NAFLD (Fig 3B). The gene and protein levels of GNMT were decreased (Fig 3C). For the PEMT reaction, PE was reduced significantly in HFHC group (p< 0.01); however, levels of PC were not changed significantly, thus increasing the PC/PE ratio significantly (p< 0.01) (Fig 3D). There were no significant concentration differences for GAA and creatine, the substrate and product for GAMT.

The products of arginine methylation via PRMT include MMA, SDMA, and ADMA. While the levels of arginine were unchanged, MMA and ADMA were significantly reduced (p< 0.01 and p< 0.05, respectively) and SDMA reduction almost reached statistical significance (p = 0.07) with HFHC diet (Fig 3E). Due to these changes, further investigation was carried out to measure the expression of PRMT1, which is responsible mainly for the methylation of MMA and ADMA [11]. While *Prmt*1 mRNA was decreased, the protein levels for this enzyme were significantly increased (p< 0.05) (Fig 3F). Circulating ADMA levels increased and approached significance (p = 0.06) (Fig 3G).

### HMG-CoA reductase DNA hypermethylation and stable global DNA methylation and hydroxymethylation with HFHC diet

**Fig 4:**
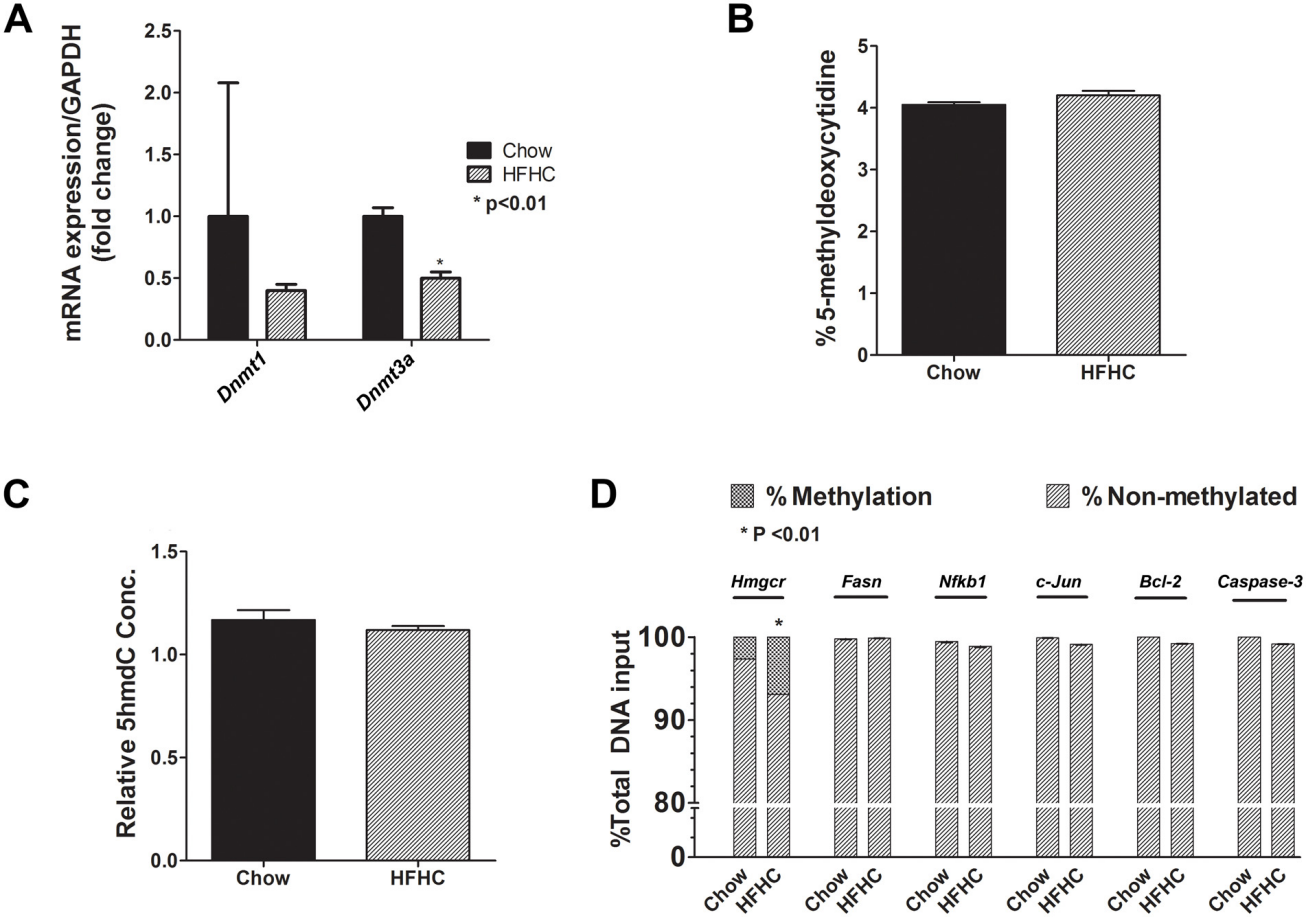
Stable global DNA methylation and hydroxymethylation and HMG-CoA reductase DNA hypermethylation in diet-induced NAFLD. (A-B) Although the gene expression for *Dnmt1* and *Dnmt3a* tended to decrease and decreased, respectively, the percent methyldeoxycytidine did not change between chow and HFHC group. (C) The relative 5hmdC concentrations also did not change. (D) Among the individual genes involved in the pathogenesis of NAFLD, *Hmgcr* was hypermethylated (p< 0.01) but there were no methylation changes for *Fasn*, *Nfκb1*, *c-Jun*, *Bcl-2*, and *Caspase 3*in HFHC group. Data are represented as mean ± SEM. Legend: B-cell lymphoma 2 (Bcl-2); DNA methyltransferase 1 and 3a (Dnmt 1 and Dnmt3a); fatty acid synthase (Fasn); 5-hydroxymethyl-2’-deoxycytidine (5hmdC); 3-hydroxy-3-methylglutaryl-coenzyme A reductase (HMG-CoA reductase, Hmgcr); nuclear factor of kappa light polypeptide gene enhancer in B-cells 1 (Nfkb1).

The methyl groups of SAM are also used by DNA methyltransferases to methylate DNA that can lead to epigenetic modifications. The status of global DNA methylation and hydroxymethylation was determined in both groups of mice. Although the mRNA expression of *Dnmt1* and *Dnmt3a*, respectively, tended to decrease or decreased significantly (Fig 4A), there were no differences in percent 5-methyldeoxycytidine between the chow and HFHC group (Fig 4B). No differences in relative 5-hydroxymethyl-2'-deoxycytidine (5hmdC) concentrations was also observed (Fig 4C). We also investigated the methylation status of individual genes, which play important roles in the pathogenesis of human NAFLD, at promoter CpG islands for lipogenic (HMG-CoA reductase, HMGCR; and fatty acid synthase, FASN), inflammatory (NFkB1and c-Jun), and apoptosis (Bcl-2 and Caspase 3) targets. There was a significant increase in *Hmgcr* methylation in HFHC group (p<0.01), whereas no methylation changes for *Fasn*, *Nfκb1*, *c-Jun*, *Bcl-2*, and *Caspase 3* were observed (Fig 4D).

## Discussion

**Fig 5:**
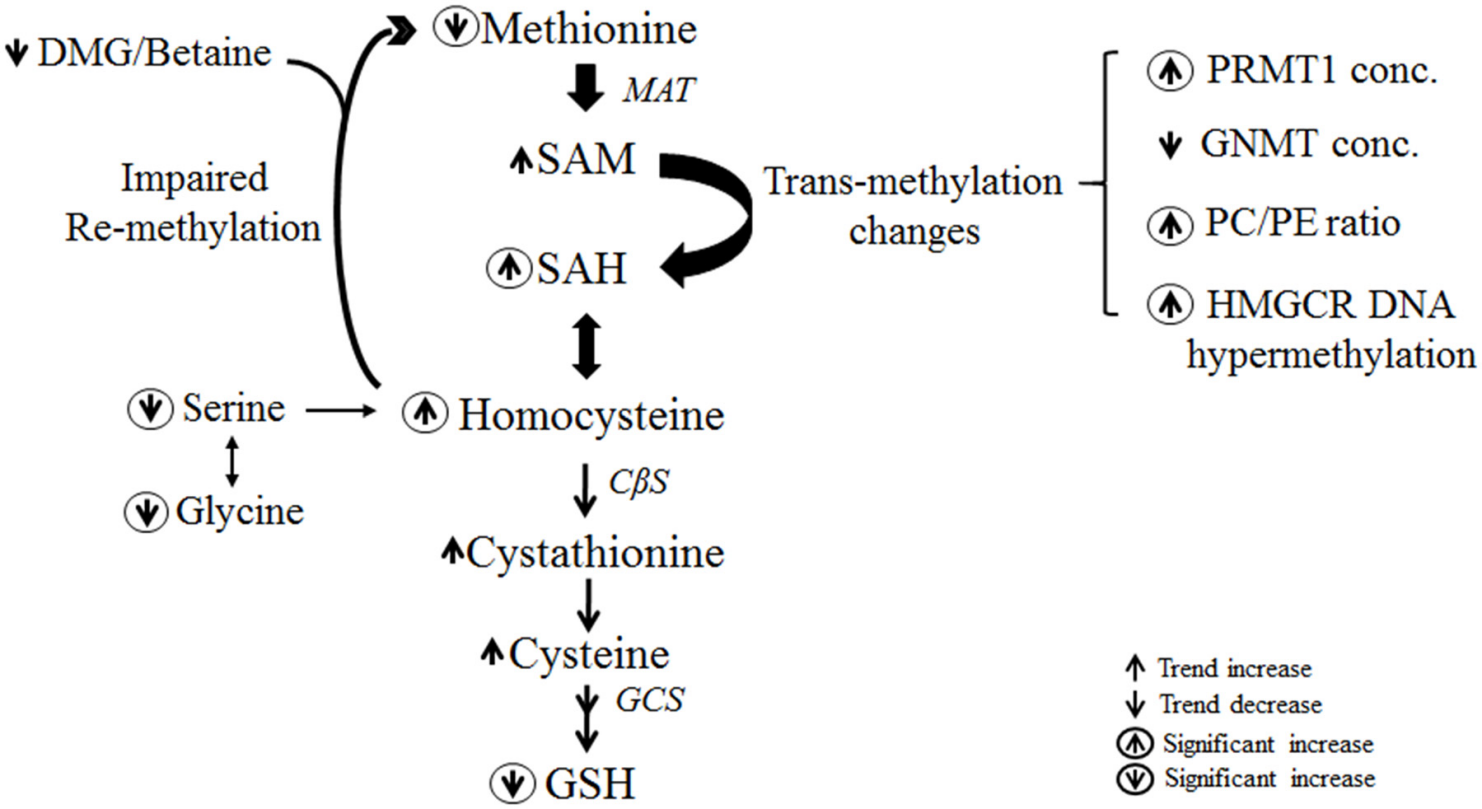
Summary of changes in the methionine cycle, transsulfuration pathway and methyltransferase reactions in advance NAFLD. HFHC diet for 52 weeks resulted in methionine depletion, excess homocysteine and aberrancy in transmethylation pathway. The decrease in the substrate serine impairs homocysteine remethylation and limits the ability to replete glutathione in the transsulfuration pathway. Legend: cystathionine β-synthase (CBS); dimethylglycine (DMG); γ-glutamylcysteine synthetase (GCS); glutathione (GSH); 3-hydroxy-3-methylglutaryl-coenzyme A reductase (HMGCR); methionine adenosyltransferase (MAT); phosphatidylcholine (PC); phosphatidylethanolamine (PE); protein arginine methytransferas 1 (PRMT1); s-adenosylmethionine (SAM); s-adenosylhomocysteine (SAH); s-adenosylhomocysteine hydrolase (SAHH).

The current study demonstrates that with long-term (52 week) feeding of a HFHC diet and development of advance NAFLD with fibrosis, there is a depletion of methionine along with an increase in its downstream products SAM, SAH and homocysteine (Fig 5). The decrease in methionine, an essential amino acid, may reflect a dietary deficiency or increase in its utilization. In the mouse model studied, the methionine concentration in the HFHC diet was higher than in chow diet (7 mg/kg vs 3 mg/kg) and it is therefore unlikely that the observed decrease reflected a dietary deficiency. It is therefore inferred that the metabolic stress imposed by a HFHC diet increases demand for methionine-derived metabolites and accounts for a decrease in hepatic methionine concentration.

The depletion of hepatic methionine stores appears to be a time-dependent phenomenon. Prior studies did not find evidence of methionine depletion after 3–12 weeks of a HFHC diet [22–25]. The methionine content of the diet was not however reported and the animals only had early stage disease with steatosis without demonstrable fibrosis. The time-dependency of the observed methionine depletion despite an adequate diet suggests that the demand for methionine is a function of disease progression. The current study and available literature do not however inform us about whether the changes in methionine metabolic pathways reflect an adaptive response to limit disease progression or actually contribute to disease progression. This is an important consideration because an adaptive response would provide a rationale for methionine supplementation whereas a maladaptive response would not. It is therefore probably wise to perform additional preclinical trials of methionine supplementation before engaging in large scale human studies.

Hyperhomocysteinemia is closely associated with the metabolic syndrome and has been shown to be present in subjects with NAFLD [14–16]. It has been demonstrated that accumulation of homocysteine activates hepatic unfolded protein response and induces oxidative stress, hepatic inflammation and fibrosis [19, 20]. Homocysteine is an intermediate in methionine metabolism, which takes place mainly in the liver [9]. The observed depletion of methionine and increase in SAM and SAH suggests increased formation of hepatic homocysteine as a result of increased transmethylation activity of SAM. However, it is important to note that the levels of SAH hydrolase decreased excessively which do correlate with SAH elevation but not the increase in homocysteine. This suggests that homocysteine accumulation results from decreased in its utilization, either from altered remethylation to methionine and/or transsulfuration activity.

Remethylation of homocysteine requires the activity of methionine synthase and betaine-homocysteine methyltransferase and the availability of betaine and serine-dependent folate cycle activity [13, 37, 38]. The current study demonstrates that the expression of these enzymes is not significantly changed under conditions of a HFHC diet. However, there is a depletion of serine which would be expected to impair transfer of methyl groups from methyltetrahydrofolate [38] and inhibit remethylation of homocysteine. The decreased dimethylglycine to betaine ratio is also supportive of the concept that impaired remethylation of homocysteine to methionine [39] contributes to methionine depletion as well as homocysteine elevation following long term administration of a HFHC diet and development of NAFLD with fibrosis. Impaired remethylation has also been suggested to be present in humans with NAFLD [21].

Providing substrates for critical enzymes in the transsulfuration pathway to replete glutathione would be expected to reduce homocysteine levels. This pathway is therefore unlikely to contribute to homocysteine elevation in NAFLD. It is already established that a high-fat diet is associated with oxidative stress and increased glutathione turnover [40–42]. In our study, there has been only a modest increase in cystathionine and cysteine levels despite a significant increase in homocysteine levels. It is known that homocysteine condenses with serine to form cystathionine, which is deaminated and hydrolyzed to form cysteine [43]. The fractional contribution of serine carbon to cysthathionine to meet the higher demands for glutathione has been demonstrated to be higher in NASH following intralipid infusion [44]. In addition, the levels of glycine, a substrate of serine, are also significantly decreased with long-term HFHC diet. In mammals, glycine is metabolized by the glycine cleavage system to form N_5_N_10_ methylene-tetrahydrofolate (THF) [45]. Catalyzed by serine hydroymethyltransferase, glycine can form serine by transfer of the methyl group from THF [44]. It is therefore inferred that availability of cystathionine and cysteine to replete glutathione stores is limited by the reduction in the substrates, serine and glycine, under conditions of long-term HFHC diet. It is imperative to explore the supplementation of these substrates to treat NASH by restoring glutathione levels and reducing the concentration of homocysteine that plays an important role in NAFLD disease progression.

It is also attractive to hypothesize that increased demand for homocysteine to replete glutathione under conditions of diet-induced oxidative stress drives the demethylation of methionine and SAM and is a key driver for methionine depletion. If so, therapies associated with decreased oxidative stress and protection of glutathione stores would be expected to ameliorate the depletion of methionine. Of note, in humans with NASH, we have observed a trend for a decrease in circulating homocysteine levels after vitamin E treatment (unpublished data from metabolomic analyses of plasma samples of subjects in the PIVENS trial) [46].

The current study also provides evidence of aberrancy in methyltransferase reactions as a result of long-term HFHC diet. GNMT is the most abundant methyltransferase in the liver and mainly involved in hepatic SAM catabolism. In *Gnmt* knockout mice, SAM becomes elevated and results in rapid development of NAFLD [47]. In the current study, SAM is only modestly elevated despite decreased GNMT levels. It is possible that SAM is markedly being utilized by other methyltransferase reactions. An increase in PEMT flux as an adaptive response to hepatic SAM accumulation is observed in GNMT knockout mice [44]. The relative increase in PC (product) to PE (substrate) ratio in the present study despite a general depletion of these phospholipids suggests continued activity of this enzyme. The depletion of these phospholipids is also observed by our group in human NAFLD [48]. The decrease in PC in particular can potentially affect membrane fluidity and activate the unfolded protein response, playing a role in the pathogenesis of NAFLD. The lack of changes in choline levels argues against increased CDP-choline mediated PC synthesis, although a definitive statement about this cannot be made.

Interestingly, the methylated arginine metabolites (ADMA and MMA) were depleted from the liver in mice after the 52 week HFHC diet. This occurred despite a significant increase in the protein levels of PRMT1, which would be expected to drive increased formation of these compounds, along with increased circulating ADMA levels. We infer this to represent increased formation of ADMA and MMA and its export from hepatocytes. It is important to note that ADMA is well known to inhibit nitric oxide synthase (NOS) activity and an important modulator of eNOS-mediated vasodilation [49, 50]. NAFLD has been associated with increased iNOS activity and inflammation and iNOS knockdown has been shown to ameliorate both inflammation and injury/apoptosis [51–54]. The depletion of hepatic ADMA in this study provides a potential mechanism for the increased iNOS observed by others under conditions of HFHC diet [52, 55]. Concomitantly, increased circulating ADMA is likely to contribute to systemic endothelial dysfunction commonly seen in NAFLD [56].

DNA methylation is an essential epigenetic mechanism and deoxycytidine methylation within CpG islands on promoters of genes inhibits transcriptional factor binding and, thus, generally inhibits gene expression [57, 58]. The development of hepatic steatosis in a mouse model was accompanied by altered expression of DNMT 1 and DNMT 3A in the liver [59]. In the current study, although the gene expression of *Dnmt3a* was significantly decreased, we did not observe any significant changes in global methylation status in mice fed with HFHC diet. Furthermore, the individual methylation of *Fasn*, *Nfkb1*, *c-Jun*, *Bcl-2* and *Caspase-3* were unchanged. Moreover, the individual methylation of FAS, NFkB1, c-Jun, Bcl-2 and caspase-3 were unchanged. Therefore, there was a lack of epigenetic adaptation to minimize de novo lipogenesis, inflammation and apoptosis in mice with NAFLD. The only potential exception is the observed hypermethylation of *Hmgcr* which likely represents an adaptive response to decrease cholesterol synthesis in the face of dietary cholesterol overload.

In summary, advanced NAFLD is associated with multiple alterations in methionine metabolism that lead to hepatic methionine deficiency and homocysteine elevation, mainly as a result of impaired remethylation of homocysteine to methionine. While high homocysteine levels provide substrate for glutathione repletion, this by itself may contribute to disease progression in NAFLD. The depletion of the substrate serine also limits the repletion of glutathione stores in response to oxidative stress. There is also aberrancy in methyltransferase reactions, as evidenced by decrease in *Gnmt* and increase in PC/PE ratio and PRMT. The decrease in ADMA and MMA levels plays an important mechanistic role in NAFLD pathogenesis by increasing iNOS-mediated inflammation and apoptosis, thus, disease progression in NAFLD.

## Supporting Information

S1 Fig Representative image of steatosis induced by a high-fat high-calorie diet for 52 weeks. Liver tissues were stained with hematoxylin and eosin (HE). In high-power field, macrovesicular steatosis and small droplet steatosis, which is centrilobular in distribution, was present in the liver under conditions of high-fat high-calorie diet for 52 weeks **(1a)**.
(TIF)

S2 Fig Representative image of inflammation and fibrosis induced by a high-fat high-calorie diet for 52 weeks. Liver tissues from mice fed a high-fat high-calorie diet for 52 weeks were stained with Masson’s trichrome stain. Scattered foci of inflammatory cells and extensive pericellular fibrosis were observed, indicating an advanced form of NAFLD.
(TIF)

S1 Table MAT, methionine adenosyltransferase; Ahcy, adenosylhomocysteinase; Gnmt, glycine N-methyltransferase; Cbs, cystathionine β-synthase; γ-Gcs, γ-glutamylcysteine synthetase; Bhmt, betaine-homocysteine methyltransferase; Ms, methionine synthase; Prmt, protein arginine methyltransferase; Dnmt, DNA methyltransferase; Gapdh, glyceraldehyde 3-phosphate dehydrogenase. (DOCX)
