# Supplementary figures and images for: Dysregulated Hepatic Methionine Metabolism Drives Homocysteine Elevation in Diet-Induced Nonalcoholic Fatty Liver Disease
Source: PLoS One. 2015 Aug 31;10(8):e0136822. doi: 10.1371/journal.pone.0136822 (PMC4556375; doi:10.1371/journal.pone.0136822)

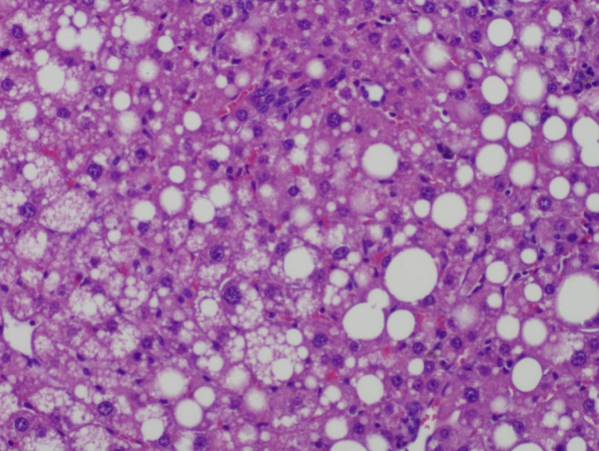

Supplement: S1 Fig — Liver tissues were stained with hematoxylin and eosin (HE). In high-power field, macrovesicular steatosis and small droplet steatosis, which is centrilobular in distribution, was present in the liver under conditions of high-fat high-calorie diet for 52 weeks (1a). (TIF) [file pone.0136822.s001.tif]

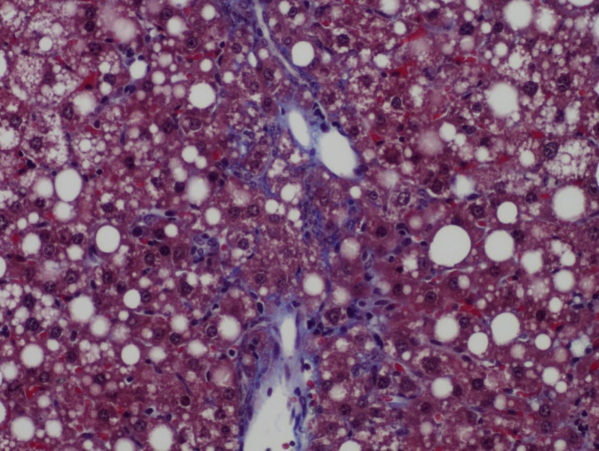

Supplement: S2 Fig — Liver tissues from mice fed a high-fat high-calorie diet for 52 weeks were stained with Masson’s trichrome stain. Scattered foci of inflammatory cells and extensive pericellular fibrosis were observed, indicating an advanced form of NAFLD. (TIF) [file pone.0136822.s002.tif]
