# Supplementary material for: Dysregulated Hepatic Methionine Metabolism Drives Homocysteine Elevation in Diet-Induced Nonalcoholic Fatty Liver Disease
Source: PLoS One. 2015 Aug 31;10(8):e0136822. doi: 10.1371/journal.pone.0136822 (PMC4556375; doi:10.1371/journal.pone.0136822)
Supplement: S1 Table — (DOCX) [file pone.0136822.s003.docx]

Supplementary Table 1. Primer sequences for Real Time reverse transcription PCR

| **PRIMERS** | **SEQUENCE** |
| --- | --- |
| mMat1A | F: 5’-CTACTGATGAGACCGAGGAATG-3’  R: 5’-CCAGGGAAGGACACCAGAG-3’ |
| mMat2A | F: 5’-GCACACCTTCAACAAGACC-3’  R: 5’-GCTTCACGAACCACTTTCTG-3’ |
| mAhcy | F: 5’-AGCTTCGTGATGAGCAACTC-3’  R: 5’-ACTCAGTAGCGGTAGTGATC-3’ |
| mGnmt | F: 5’-GCCTACGTTCCCTGCTACTT-3’  R: 5’-CCACATCTGCACCCAAATGC-3’ |
| mCbs | F: 5’-ACAGGG ATCGCCAGAAAG-3’  R: 5’-GGATGAAGTCGTAGCCAATC-3’ |
| mγ-Gcs | F: 5’-CTACGGAGGAACGATGTC-3’  R: 5’-TTCTGGCAGTGTGAATCC-3’ |
| mBhmt | F: 5’-TCCATTGTCGGCGTGAAC-3’  R: 5’-GGCTGGCTCATCAGGTAAG-3’ |
| mMs | F: 5’-GACTCATCACTCCATCCT-3’  R: 5’-TGCTCCTCCTATCAACAA-3’ |
| mPrmt1 | F: 5’-TTCACAATCGGCATCTCTTC-3’  R: 5’-GACAATCTTCACAGCATAATCG-3’ |
| mDnmt1 | F: 5’-AGTGCAAGGCGTGCAAAGATATGG-3’  R:5’- TGGGTGATGGCATCTCTGACACAT-3’ |
| mDnmt3A | F: 5’-ACAAGAATGCTACCAAAGCAGCCG-3’  R: 5’-TGAGAACTTGCCATCTCCGAACCA-3’ |
| mGapdh | F: 5’-AGAAACCTGCCAAGTATGATG-3’  R: 5’-GGAGTTGCTGTTGAAGTCG-3’ |

# MAT, methionine adenosyltransferase; Ahcy, adenosylhomocysteinase; Gnmt, glycine N-methyltransferase; Cbs, cystathionine β-synthase; γ-Gcs, γ-glutamylcysteine synthetase; Bhmt, betaine-homocysteine methyltransferase; Ms, methionine synthase; Prmt, protein arginine methyltransferase; Dnmt, DNA methyltransferase; Gapdh, glyceraldehyde 3-phosphate dehydrogenase.
